# A Nubian Complex Site from Central Arabia: Implications for Levallois Taxonomy and Human Dispersals during the Upper Pleistocene

**DOI:** 10.1371/journal.pone.0069221

**Published:** 2013-07-24

**Authors:** Rémy Crassard, Yamandú Hieronymus Hilbert

**Affiliations:** Centre National de la Recherche Scientifique CNRS, Maison de l'Orient et de la Méditerranée, UMR 5133 ‘Archéorient’, Lyon, France; IPATIMUP (Institute of Molecular Pathology and Immunology of the University of Porto), Portugal

## Abstract

Archaeological survey undertaken in central Saudi Arabia has revealed 29 surface sites attributed to the Arabian Middle Paleolithic based on the presence of Levallois blank production methods. Technological analyses on cores retrieved from Al-Kharj 22 have revealed specific reduction modalities used to produce flakes with predetermined shapes. The identified modalities, which are anchored within the greater Levallois concept of core convexity preparation and exploitation, correspond with those utilized during the Middle Stone Age Nubian Complex of northeast Africa and southern Arabia. The discovery of Nubian technology at the Al-Kharj 22 site represents the first appearance of this blank production method in central Arabia. Here we demonstrate how a rigorous use of technological and taxonomic analysis may enable intra-regional comparisons across the Arabian Peninsula. The discovery of Al-Kharj 22 increases the complexity of the Arabian Middle Paleolithic archaeological record and suggests new dynamics of population movements between the southern and central regions of the Peninsula. This study also addresses the dichotomy within Nubian core typology (Types 1 and 2), which was originally defined for African assemblages.

## Introduction: The Arabian Middle Paleolithic

Comprehensive studies of the Arabian Middle Paleolithic are relatively recent, compared to the more established research traditions of Europe, south and east Africa or the Mediterranean Levant. In recent years, however, the Arabian Peninsula has experienced a considerable expansion of field research, aimed at the categorization of its prehistoric lithic assemblages and the investigation of its place in both human evolution and human dispersal events out of Africa [1]–[20].

The Middle Paleolithic lithic assemblages found in Arabia are mainly characterized by the presence of the Levallois technology *sensu lato*. This specific reduction strategy is defined by the production of blanks showing predetermined dimensions and shapes. This predetermination is achieved by diverse variations in core volume preparation [21]–[26]. Dated Levallois occurrences are known from various parts of the Arabian Peninsula, namely southwestern Yemen [6], [16], the Emirate of Sharjah [11], southern Oman [12], [20] and northern Saudi Arabia [14], [15], where different types of Levallois reduction have been observed among Arabian Middle Paleolithic assemblages [27]–[29].

The discovery of the typically northeast African Nubian Levallois technology in southern Arabia represents a clear technological connection between northeast Africa and the Arabian Peninsula [12]. In Arabia, Nubian technology was initially identified in southwest Oman [12] and attributed to the Nubian Complex of Dhofar. Prior to this, comparable cores had been found in Hadramawt, eastern Yemen [5], [6], [27], [30], however, due to sampling constraints in Yemen and the lack of chronological control over these surface assemblages, researchers remained impartial as to whether these cores were related to Levallois-based industries from Africa or the Levant. Preliminary analyses from Yemen supported a connection between South Arabia and the Levant, however, chronological and technological data from Dhofar now suggests an introduction of the Nubian reduction method through the Southern Dispersal Route. Researchers working on the lithic samples from Jebel Faya NE-1, United Arab Emirates, have detected a general affinity between Assemblage C and East African Middle Stone Age (MSA) industries [9], [11]. Additionally, Middle Paleolithic occurrences from Shi'bat Dihya, southwest Yemen have yielded samples which indicate a lithic industry anchored within a local tradition [16], [17]. The assemblages from Shi'bat Dihya suggest a propensity towards the production of elongated blanks by the use of varying single platform unidirectional reduction schemes (*tournant/semi-tournant* and “frontal” *débitage*), although a smaller contribution of Levallois-like flake production also was noted. Middle Paleolithic industries found in the Jubbah area of Northern Saudi Arabia [14], [15] present a greater affinity, both typologically and technologically, with Levantine assemblages. These are characterized by a Levallois preferential blank production with centripetal preparation, indicative of Tabun C-type assemblages [31], [32], along with a Levallois unidirectional convergent technology, typical of Tabun B-type. The variability observed within the lithic assemblages from Arabia, therefore, shows different traditions that likely reflect different populations that inhabited the Arabian Peninsula during the second half of the Late Pleistocene, adding to the complexity of the prehistoric record of the Peninsula.

**Figure 1:**
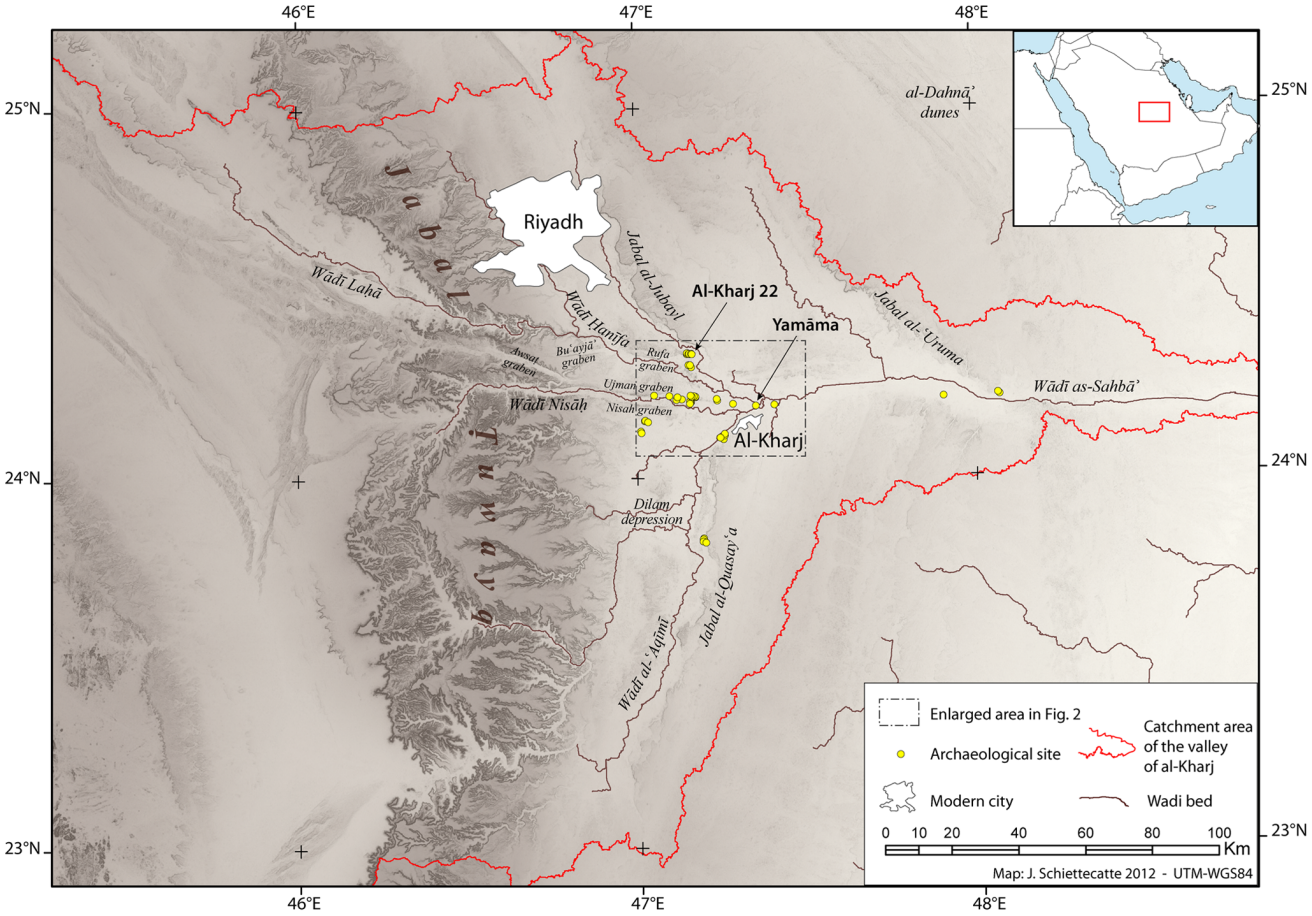
Topographic map of the Al-Kharj region in central Saudi Arabia. Spots are showing archaeological sites discovered during the 2011 survey activities. Map by J. Schiettecatte, CNRS.

**Figure 2:**
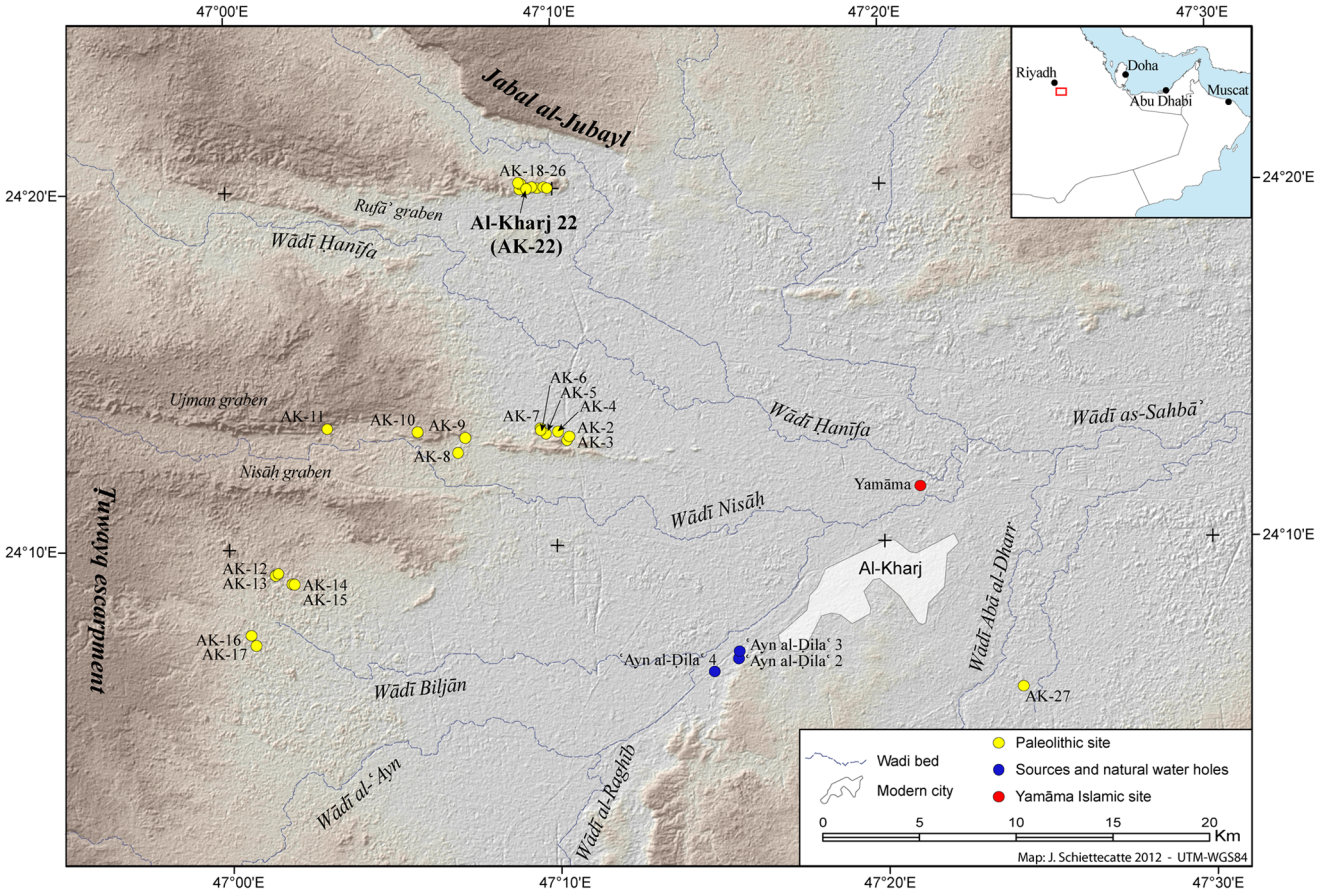
Survey activities undertaken in 2011. In the proximity of the modern town of Al-Kharj, with the discovered Middle Paleolithic surface scatters, including Al-Kharj 22 site. Map by J. Schiettecatte, CNRS.

To expand and enhance the growing data set of Arabian Paleolithic sites, a Saudi-French archaeological project was initiated in 2011, under the direction of Dr. Jérémie Schiettecatte (CNRS, Ivry-sur-Seine, France) and Prof. Abdulaziz al-Ghazzi (King Saud University, Riyadh, Saudi Arabia). A detailed field survey was undertaken in the proximity of the modern town of Al-Kharj, central Saudi Arabia (Figure 1) which revealed a total of 29 Middle Paleolithic surface scatters (Figure 2). Here we present the results from the archaeological investigation at Al-Kharj, focusing on lithic technology and the interpretation of the Levallois methods. In particular, the Nubian Levallois Method will be discussed more explicitly, given its distribution across both North Africa and Southern Arabia.

## Lithic Technological Analysis

### The Levallois definition and its use in Arabia

Middle Paleolithic/Middle Stone Age (MP/MSA) sites are often characterized by the Levallois methods of blank production, although some exceptions are noted. In Africa, Levallois technology remains a part of the Late Stone Age technological repertoire [33]–[36]. Similarly, Levallois methods are present in some European Early Upper Paleolithic assemblages [37]–[40], and also in Near-Eastern Holocene assemblages [41]. These cases, however, are exceptions given that the majority of MP/MSA sites recognized across Europe, Middle East and Africa have the Levallois blank production as the main technological element [24], [42]–[50].

The Levallois reduction methods are marked by the production of blanks with predetermined shapes (flakes, blades, points) using different methods of flaking (*débitage*), which can be recognized through the study of reduction patterns, or *chaîne opératoire* [24], [25]. The Levallois technology, also understood as a concept [26], [51], was widely described and illustrated through the study of various archaeological assemblages [21], [52]–[54] and experimental data [55], [56]. Characteristic for this type of reduction is a hierarchical use of core surfaces. The dorsal surface, from which the Levallois blanks are removed, is termed the working surface or Levallois surface, while the ventral surface is called the platform surface, as this is from where the preparation of the dorsal surface takes place. Levallois cores are often asymmetric in cross-section due to the arrangement of these surfaces, which undergo different treatment across the reduction phases. Variability within the Levallois reduction is primarily expressed by the diversity with which prehistoric flint-knappers prepared the Levallois surface; an aspect that influences the shape of the desired end product. Equally characteristic is the preparation of a preferential striking platform ([57]: 112) by the removal of flakes from the ventral surface, which leads to the faceted butts seen on general Levallois flakes and more specifically, on the *preferential* Levallois blanks.

Arabian lithic assemblages containing Levallois technology have been known since the 1930′s from surface occurrences found in Yemen, namely in the Hadramawt region [6], [27], [30], [58]–[62]. More recently, stratified sites found in the Tihama/al-Mahwit region (Wadi Surdud) [16], [17], [28] have revealed the chronological range and technological variability of this reduction method in Southwestern Yemen. Elsewhere in the Arabian Peninsula, assemblages with Levallois technology have been amply recognized [63], [64]. Stratified and dated Levallois occurrences are reported from Aybut Al Auwal in the Dhofar region of Oman, in the United Arab Emirates at Jebel Faya NE-1 and in Saudi Arabia within the Jubbah region. These discoveries have yielded dates provided by the Optically Stimulated Luminescence (OSL) method. In Dhofar, one Nubian Levallois core and approximately 10 flakes and blades have been dated to 106.6±6.4 thousand years ago (kya) [12], while the layers containing Assemblage C at Faya NE-1 have yielded three different dates (127±16, 123±10, and 95±13 kya) falling early within Marine Isotope Stage (MIS) 5 [11]. The identification of Levallois reduction in Assemblage C, however, remains uncertain due to the limited number of core samples. In Saudi Arabia, within the Jubbah region, sites containing Levallois technology have shown a substantial chronological range spanning MIS 7 (211±16 kya), MIS 5c (95±7 kya), and MIS 5a (75±5 kya) [14], [15]. Noteworthy here is the possible use of Levallois technology within the lower levels from Jebel Qattar JQ-1, which may represent the oldest manifestation of Levallois reduction in Arabia (MIS 7). An additional Levallois assemblage from the Jubbah area has been excavated at the Jebel Katefeh JKF-1 site, where surface and buried lithics have been associated with sediments dating to the MIS 5a-b (90–85 kya) [15].

Among these dated assemblages, the samples from Dhofar are of particular interest given the chronology they provide for the Nubian presence in Arabia. This highly standardized method of blank production represents a technological procedure that aims at the manufacture of triangular flakes and blades, which we regard as technological marker with a high recognition value, due to the either bidirectional, centripetal or bidirectional/centripetal scar pattern on its dorsal surface.

### Levallois point production and Nubian Levallois reduction

Bordes initially described the Levallois point production based on assemblages found in northern France and Jordan [21]. In 1980, he individualized two types of Levallois point cores: the unidirectional convergent and unidirectional divergent Levallois point cores, both of which represent a *preferential* Levallois blank production (vs. recurrent Levallois methods). While the Levallois surface on a unidirectional convergent core is prepared by removals struck from the same striking platform as the preferential removal, unidirectional divergent cores are prepared by removals taken from a striking platform arranged opposite to the preferential striking platform [22]. Bordes [22] also discusses the Nubian cores described by Guichard and Guichard [65], which are separated into two types. The Type 1 is a “Levallois point core characterized by a special technique”, which Bordes defined as a Levallois point core prepared by two unidirectional divergent removals undertaken from the distal part of the core. Type 2 cores are marked by an elaborated centripetal preparation arranged perpendicularly to the central axis of the triangular silhouette of the Levallois surface from which a Levallois point, unlike the “classical” Levallois points ([65]: 68–69), is struck. Guichard and Guichard [65] did not consider the objective of this second scheme as a Levallois point *sensu stricto*, given that the preferential removal does not follow a central guiding ridge. Both studies, however, conclude that the product of this reduction is a triangular Levallois flake [22], [65].

Technological analyses undertaken on Nubian Complex sites in Egypt and north Sudan considerably add to the discussion of the dichotomy among Nubian cores [24], [35], [54], [66]–[69]. While previous researchers concentrated on the taxonomic classification of these cores into types based primarily on morphological characteristics, the technological approach has illuminated how Nubian cores were reduced. Following Van Peer's amendments to the Nubian Type 1 category [24], preparation of the Levallois surface is achieved through the detachment of two distally divergent *débordant* elements from a distal platform. This particular preparation aims at the creation of a centrally placed distal to medial ridge, while the medial to proximal portion of the Levallois surface is prepared by centripetal removals [24]. Concerning the pattern of preparation involved in the Nubian Type 2 cores, Van Peer and colleagues conclude: “the pattern of preparation itself grades between that of the classical Levallois method and the Nubian Type 1 method” ([54]: 50). This means that Type 2 Nubian cores sometimes present a very short distal ridge mirroring the technical gesture undertaken when preparing a Nubian Type 1 Levallois surface, blurring the boundary between the two core types.

Chiotti et al. [67] offer an alternative perspective to Nubian core typology (Type 1 and Type 2). Using refittings and quantitative analysis, the authors show how the Nubian Type 1, Type 2 and ‘classical centripetal’ Levallois cores may represent separate stages within a continuous centripetal Levallois reduction. Indeed, Chiotti et al. [67] propose that by removing the distal portion of a Nubian core, such differences are muted. At this point the authors refer to van Peer [70] who coined the term ‘Safaha Method’, which describes an additional step in the Nubian preparation schema identified at Nazlet Safaha 1 and 2. The protruding distal/central guiding ridge created by the distal diverging preparation is removed by a blank struck from the distal platform. These are, to a greater extent, elongated blanks presenting expanding lateral edges (fan shaped); Safaha blanks were not further modified into tools and served the re-preparation of Levallois surface with the preferential end-product being a “double-pointed flake” ([54]: 50). Chiotti et al. interpret the “Safaha flake” [67] as a transformation element that modifies a triangular Nubian core into an oval Levallois core, further obscuring the identification of core types.

**Figure 3:**
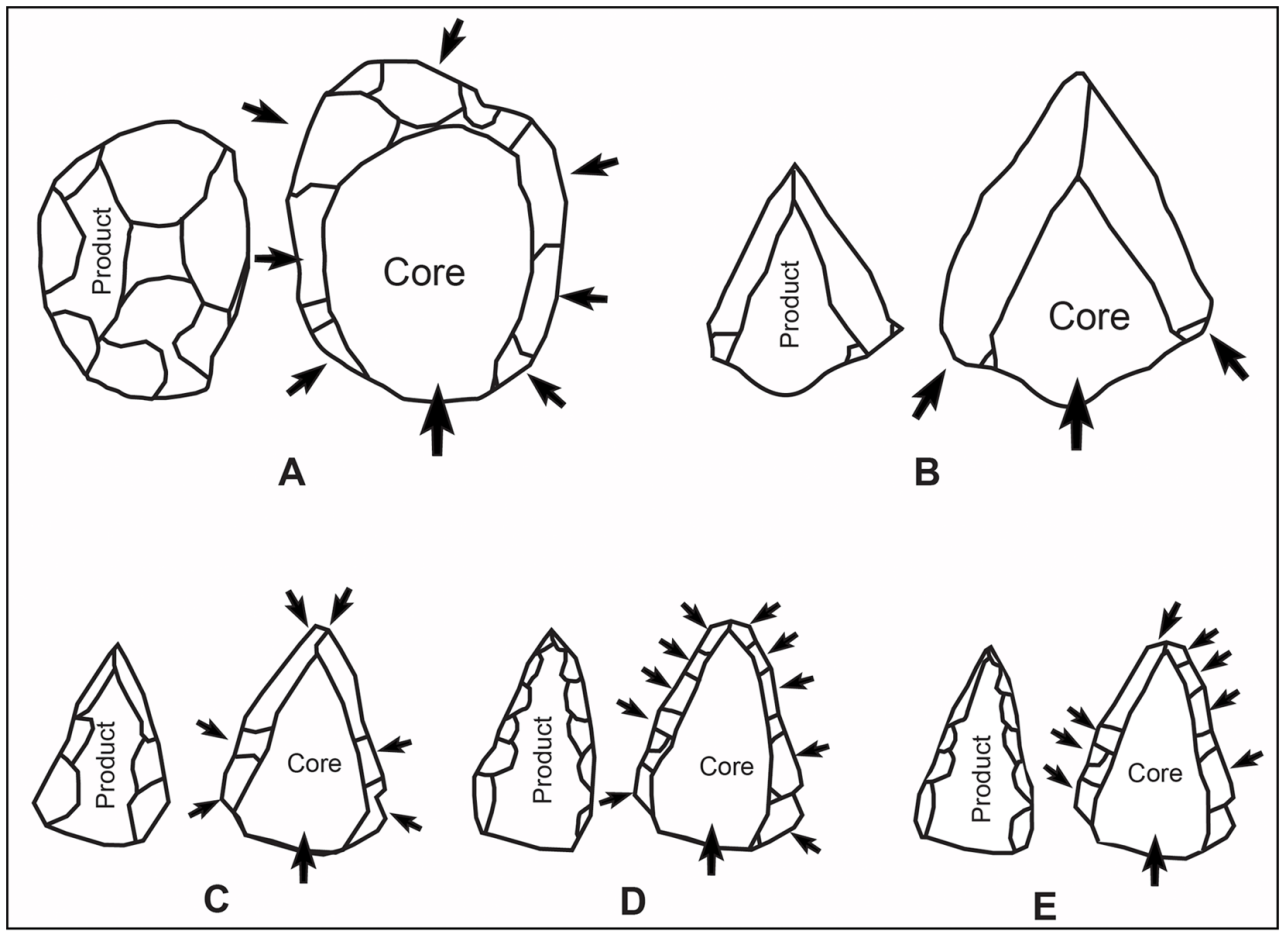
Levallois methods schemata: figuration of product and core shapes for each method. A: Preferential Levallois flake production with centripetal preparation; B: Preferential Levallois point production with unidirectional convergent preparation; C: Nubian Levallois type 1 with distal divergent preparation; D: Nubian Levallois type 2 with double lateral preparation; E: Nubian Levallois type 1/2 with mixed type 1 and type 2 preparation.

Concerning the plasticity within the Nubian technology and the interchangeability between the Nubian Type 1 and Type 2 cores, Chiotti et al. ([67]: 316) argue in favor of condensing these preparation methods into a general Nubian technology. This aspect of Nubian blank production was further articulated by Usik et al. [20]. Technological analysis of Nubian assemblages from Dhofar indicates that the overlap between the preparation methods, which culminate in the shaping of Nubian Type 1 and Type 2 cores, may be identified as “Nubian Type 1/2” ([20]: 7) (Figure 3). This plasticity in core dorsal surface preparation was also acknowledged by Crassard and Thiébaut [27], who differentiate five methods of point production based on Nubian cores from the Hadramawt region in Yemen.

For this study, we summarize the typo/technological characteristics that make up the Type 1, 2 and 1/2 cores under the rubric Nubian technology. The further discrimination between these core types within the Nubian assemblages is deemed unnecessary; we will refer to the specific preparation types previously associated with the core types (Type 1, 2 and 1/2). The site and data presented here gives insights into a largely un-sampled region of the Arabian Peninsula. The technological background to the identification of technological units discussed above serves as a guide for approaching surface site assemblages pertaining to the Paleolithic period found in central Saudi Arabia.

## Geomorphological and Climatological Framework and Site Location

The area surrounding the city of Al-Kharj, situated ca. 70 km southeast of Riyadh, is marked by a variety of geomorphological features including structural scarps, inselbergs, a complex drainage network, alluvial fans, outwash plains and sand dunes [71], [72]. The convergence of the Central Arabian Graben system comprised of the Nisah, Awsat, Bu‘ayja’ and Ujman, Rufa and the Mugharah grabens greatly influences the local geomorphology. While a succession of northwest oriented scarps marks the northern part of the studied area, the southern scarps are oriented towards the southwest (Figure 2). This change of orientation and the dip of the cuestas are caused by the formation of the Central Arabian Arch, which is related to the Late Tertiary to Quaternary upwarp associated with the Red Sea rifting [71], [73]. The locally exposed lithology is composed of Late Jurassic and Cretaceous formations which are partially blanketed by diverse Quaternary sediments of both aeolian and fluvial morphogenesis.

The flow direction of the wadis in the area is dictated by the homocline of the Arabian Shelf and the Central Arabian Graben System. The main riparian systems in the region run from the Tuwayq Escarpment, which rises abruptly to the west of the Al-Kharj area, across the Tuwayq Plateau in a general west-east course, thus serving as a conduit between the escarpment parallel to the Red Sea and the interior of the Arabian Peninsula. Towards the center of the Al-Kharj area, the wadi courses follow the aforementioned graben structures [71].

As part of the Saharo-Arabian desert belt, the climate of central Saudi Arabia is tied to the climate systems of the Mediterranean and the African/Asian monsoon. While the north receives moisture associated with winter cyclones driven by subtropical jet streams from the Mediterranean (northwesterly), the south is predominantly influenced by the summer incursion and northward migration of the Intertropical Convergence Zone (ITCZ). This atmospheric shift brings the Indian Ocean monsoon to the south of the Arabian Peninsula [74]–[76]. Additionally, recent studies [77], [78] have shown that enhanced African monsoon circulation may have also delivered increased precipitation to northern and central regions during the Late Pleistocene.

Paleoclimatic data for central Saudi Arabia indicating pluvial conditions is at present, somewhat sparse. Fluvial sediments identified near the foot of the Tuwayq Escarpment have been dated to ca. 54 and 39 kya and provide some indication of increased rainfall during MIS 3 in the general area [79]. There are, however, a considerable number of records which attest to the changing climatic conditions across Arabia during the Late Pleistocene, e.g. [74]–[85]. Humid periods marked by increased rainfall, are known to have occurred during MIS 7, 5e and at the onset of the Holocene. Pluvial conditions associated with MIS 3 have also been noted throughout the Arabian Peninsula and are represented by channel activation and lake formation [79], [83]–[85]. Whilst radiocarbon age estimates for this period [83], [84] remain questionable, OSL-dated deposits indicate that increased humidity occurred at ca. 55 and 39 kya [79], [85]. It is clear, therefore, that favorable environmental conditions during times of increased rainfall would have facilitated the expansion and development of human groups during the Late Pleistocene.

**Figure 4:**
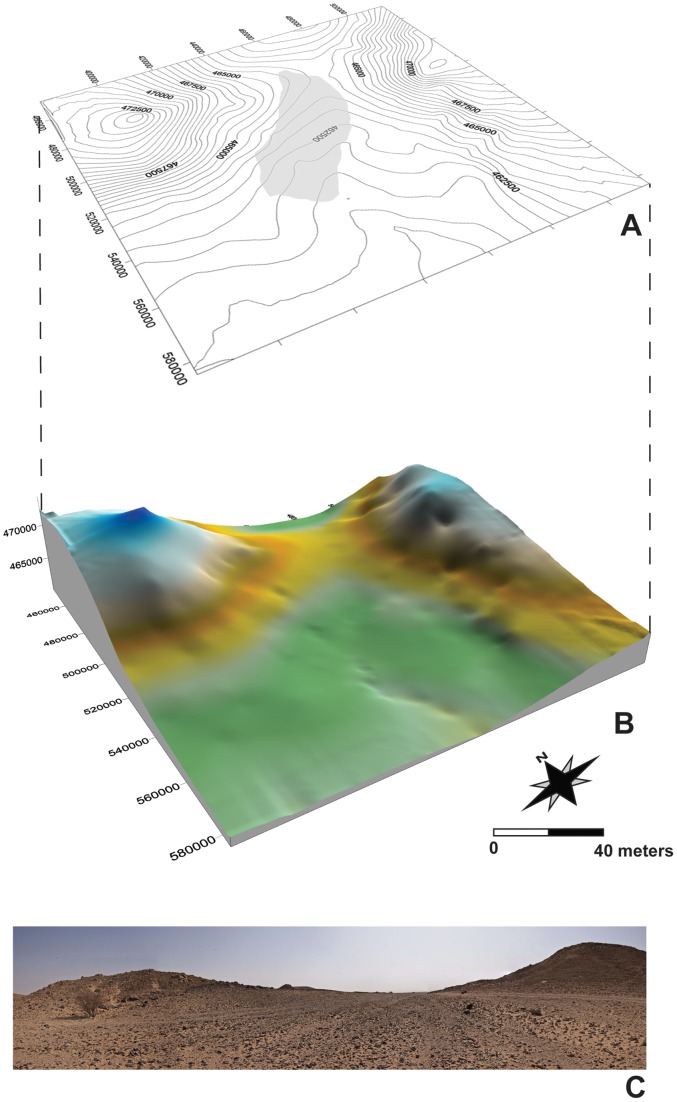
Al-Kharj 22 site. A: topographic map with isolines, the grey area within the plot indicates the extent of the surface scatter; B: orographic map with slightly exaggerated reliefs; C: panoramic view of the site from the South-West.

The surface scatter of Al-Kharj 22 (or AK-22) is situated at the eastern portion of the southern fringe of the Rufa Graben (N 24.33348°, E 47.1537°); within the Ashqar Maraghah segment. The surface site is approximately 20×60 m and lies on a slight east to west slope, flanked by inselbergs on its northern and southern faces (Figure 4). A low plain filled with recent aeolian sediments dissected by small erosional gullies characterizes the site's surroundings. Dhugum member cuestas are visible to the northeast, east and southeast of Al-Kharj 22. The lithology surrounding the site is marked by the contact of the Sulaiy limestone, the Cretaceous Dhugum member and Yamamah formation. These formations have been recognized as the source of the locally used lithic raw materials across the majority of identified sites. At Al-Kharj 22 only the Dhugum member outcrops are visible, while the closest Yamamah formation outcrops are located ∼6 km to the west and east of the site. The Dhugum outcrops, which are embedded within the lower portion of the fine- to coarse-grained and beige to brown sandstone of the Biyadh formation, are characterized by secondarily ferruginized and silicified coarse- to fine-grained white or brown quartzites. These are the most common raw material used at the Al-Kharj 22. To a lesser extent, violet silicified siltstones of unknown provenience were also observed.

The site is composed of blank production debris with no identifiable zonations within the surface scatter. Artifact concentration within the well-delimited scatter ranges between high (more than twenty artifacts per square meter) to moderate density (between five and twenty artifacts per square meter). Cores, blanks and tools have been incorporated into a 10 to 30 centimeters thick carpet composed of aeolian sediments and small to medium sizes clasts, which have been affected by taphonomic processes. This postdepositional displacement is evident on the diversely patinated artifacts found within the aforementioned sediments. Artifact patination is reliant on raw material properties and the milieu in which the objects were deposited [86]–[88]. At Al-Kharj 22, artifacts present severe surface modifications in the form of desert varnish (*sensu* [89]), which is accompanied by rounded edges. Alternatively, some artifacts show little modification other than slight change in coloration. In the latter case, negative ridges and artifacts edges remain sharp. Unfortunately, absolute dates, which would provide a chronological context for the lithic assemblage of Al-Kharj 22, could not be obtained. Further work on dating the site is planned during forthcoming field work

### Considerations on the sampling methodology and general remarks on the Al-Kharj 22 assemblage

In arid environments, which are largely characterized by sediment erosion and subsequent displacements rather than by deposition and stabilization [5], [67], [90], [91], surface artifacts scatters are the most common source of archaeological data. As with any other surface site, Al-Kharj 22 is susceptible to a series of factors that may bias both the constitution and associated interpretations of the collected samples. Primary among these factors is the inability to absolutely date surface assemblages and securely assess the unity of the collected samples to a specific depositional event. Given that the majority of surface scatters identified across the Arabian Peninsula are located in the vicinity of prominent raw material sources [2], [5], [12], [92], [93], these habitually form across considerable chronological depth, thus often presenting a mixture of diverse typological and technological elements pertaining to diverse lithic industries. While lithic industries are defined, among other elements, by technological and typological features with a set range of frequencies within regional and chronological frames [94], [95], correlations between surface assemblages and any lithic industries in particular have to be viewed as tentative. Moreover, artifact frequencies within such assemblages may be biased by the sampling strategy, making the use of artifact counts and their expression as percentages of little significance for the categorization and subsequent cultural affiliation of these assemblages. A more effective method for analyzing such scatters is to adopt a selective sampling strategy that encompasses diagnostic elements, which are subsequently examined from a technological and taxonomic perspective [2], [5], [6], [19], [93]. The technologically diagnostic elements found within surface scatters can then be compared to industries exhibiting analogous lithic production methods found within dated contexts. It is this more robust strategy that has been employed for the sample studied at Al-Kharj 22.

**Figure 5:**
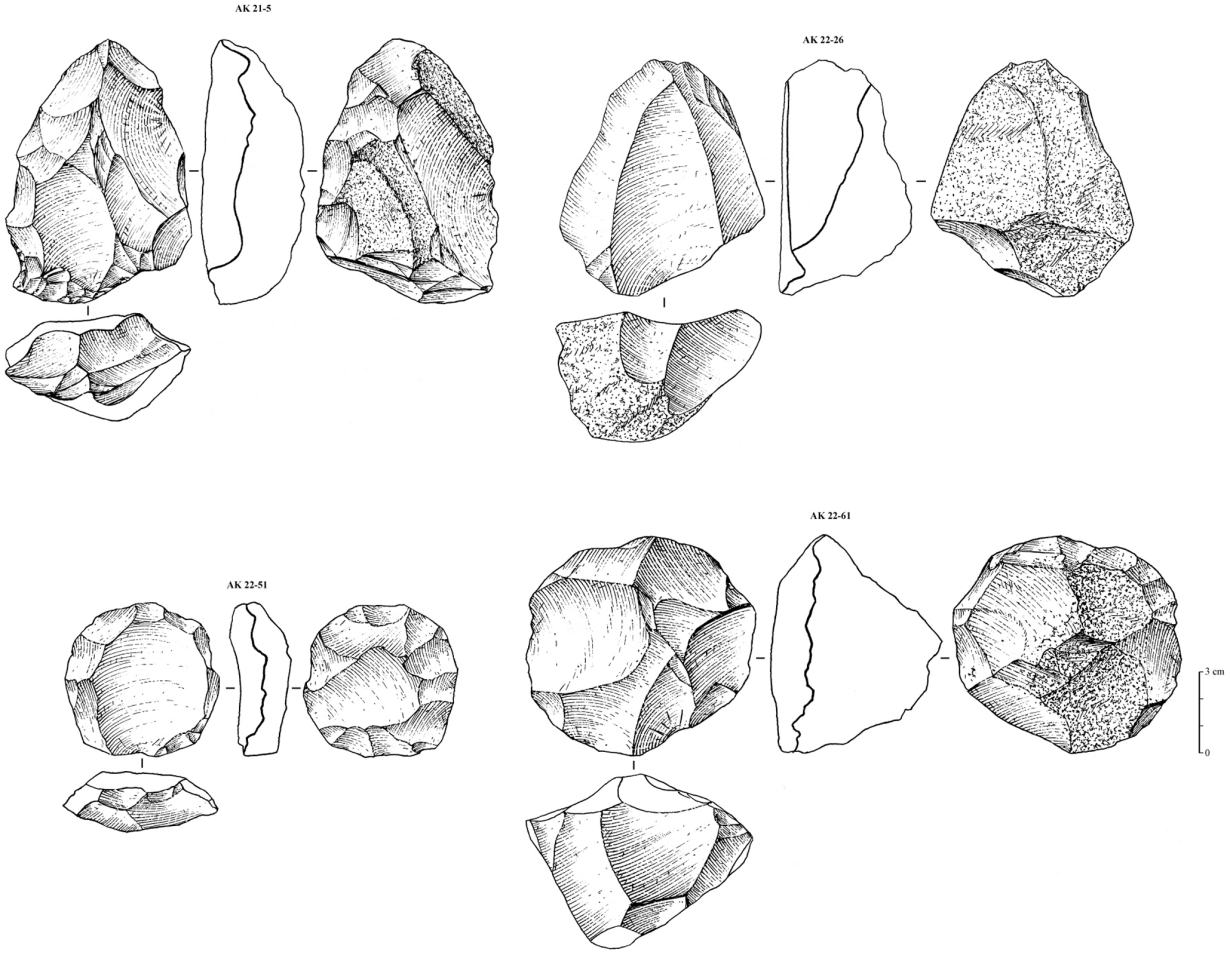
Levallois cores from Al-Kharj 22, non-Nubian. AK 21–5: non-preferential Levallois core, recurrent centripetal, maybe a prepared Nubian core or an abandoned one (from Al-Kharj 21 site considered as a northern extension of Al-Kharj 22 site); AK 22–26: Preferential Levallois core with unidirectional convergent preparation; AK 22–51: Preferential Levallois core with centripetal preparation; AK 22–61: Recurrent centripetal non-preferential Levallois core. Drawings by G. Devilder, CNRS.

A total of 177 artifacts encompassing cores and blanks were collected and analyzed (Table 1). Of these, 123 are cores that have been categorized and analyzed according to the diverse technological reduction schema employed. Levallois and non-Levallois blank production have been identified at the site (Figure 5). Levallois preferential cores with centripetal preparation, Levallois preferential point producing cores, Levallois recurrent and cores generally attributed to the Levallois concept (*sensu* [26]) have been identified within the sample. The Nubian Levallois production component found within the Al-Kharj 22 sample will be discussed separately in the following sections.

**Table 1:** Total artifact counts for the Al-Kharj 22 collection sample.

Cores	number
Nubian Levallois	16
Levallois centripetal recurrent/radial	8
Levallois preferential General	56
Levallois preferential with centripetal preparation	9
Levallois preferential point	1
Two unopposed platform core	1
Bidirectional opposed	6
Single platform flake core	7
Levallois preform	19
**Debitage**	
Cortical *débordant*	6
*Débordant* element	1
Levallois *débordant*	5
Levallois flake	23
Levallois preferential flake	4
Flake	15
**Total**	**177**

Single platform unidirectional cores and bidirectional cores have also been identified. They present unprepared or simple facetted (maximum of three removals) striking platforms. Reduction directionality observed on the single platform cores is exclusively unidirectional; the core's working surface is placed perpendicular to the striking platform and reduced in a flat fashion (*débitage facial*, [96]). Bidirectional double-platforms cores have either two opposed platforms with intersecting working surfaces or unopposed platforms with non-intersecting working surfaces. In the case of bidirectional opposed cores, platforms are used free of hierarchy, meaning that both platforms have received equal treatments and were used to the same degree to produce blanks in a recurrent mode.

The analyzed blanks present attributes consistent with Levallois and non-Levallois reduction modalities. These encompass a variety of *débordant* elements reduced from the peripheries of the core's working surface in order to maintain or create convexities [21], [22], [23], [26]. Two types of *débordant* elements have been identified; namely Levallois and cortical *débordants*. Whereas Levallois *débordants* exhibit scars from the preparation of the ventral and dorsal surfaces of the cores [12], [20], [24], [45], [47], [96], cortical *débordants* present a cortical back indicating the absence of ventral preparation [93], [97]. Preferential Levallois flakes, which are end products of a preferential Levallois reduction, and Levallois debitage, encompassing blanks attributed to the Levallois *sensu lato* reduction, were also found in the assemblage.

As such, the appearance of the Al-Kharj 22 assemblage fits comfortably within the general pattern of the Middle Paleolithic of Arabia [6], [8], [64], [98]. One specific technological aspect of the assemblage, however, makes the Al-Kharj 22 sample unique, namely the presence of the Nubian method of Levallois reduction. For the purpose of illustrating the occurrence of the Nubian methods within the Al-Kharj 22 assemblage, detailed technological descriptions will be given for the 16 cores attributed to these specific reduction modalities.

## Results: Nubian Production System from Al-Kharj 22

**Figure 6:**
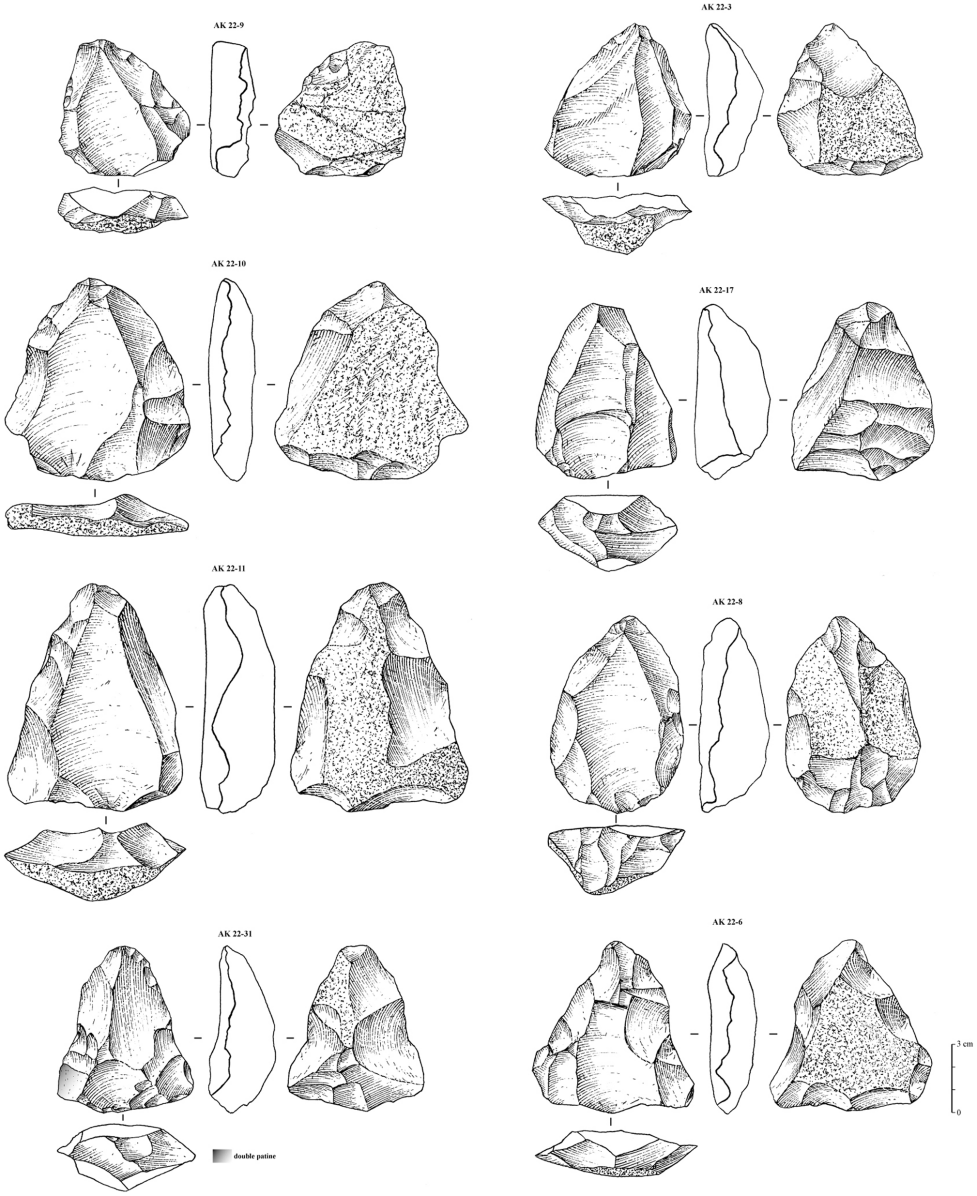
Nubian preferential Levallois cores from Al-Kharj 22. Drawings by G. Devilder, CNRS.

**Figure 7:**
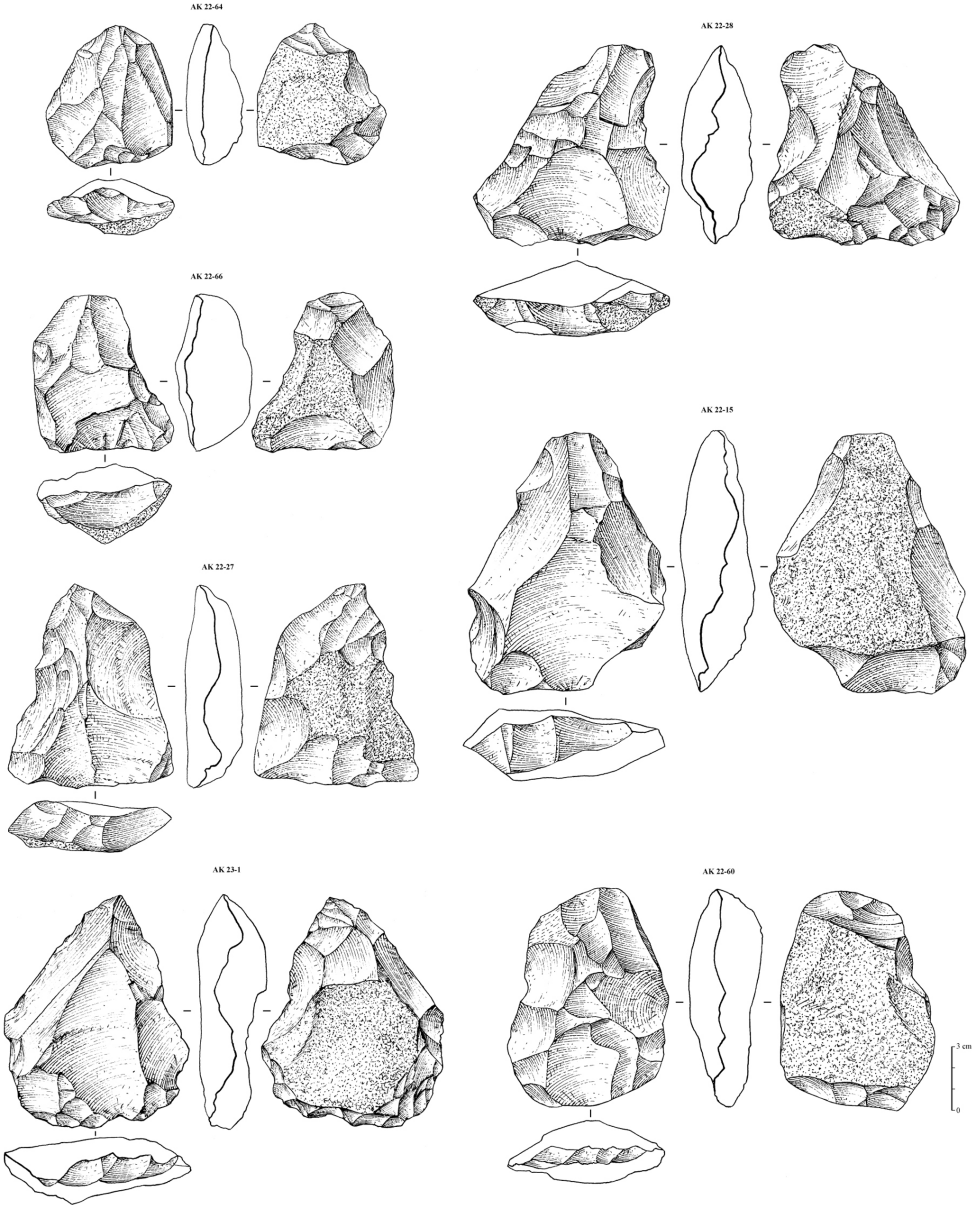
Nubian preferential Levallois cores from Al-Kharj 22. Drawings by G. Devilder, CNRS.

The 16 Nubian cores from Al-Kharj 22 were studied using technological diacritic schemes that show the reduction and preparation of the Levallois surface on the cores, whilst quantitative analysis was also undertaken. Based on these diacritics and attribute analysis all previously discussed types of Nubian preparation could be identified. The cores presenting Type 1 preparation exhibit a well developed centrally placed distal to medial guiding ridge, while the proximal portion of the core exhibits either centripetal or unidirectional scars (Figures 6, 7). The cores with a Type 2 preparation present predominantly centripetal preparation and short distal ridges prepared by either short distal diverging removals or distally converging removals dealt from a well developed distally placed supplementary platform. This particular aspect of Levallois surface maintenance has been observed on all cores generally attributed to the Nubian methods of reduction and represents one of its main characteristics. Regardless of the preparation method (Type 1, 2 and 1/2), core shape was exclusively triangular to sub triangular. Metrically, the Nubian Levallois cores average 84.29 cm in length, 66.91 cm in width and 28.51 cm in thickness (core measurements were taken with the specimens oriented accordingly to their technological axis). The largest specimen measured 125.2 cm in maximum length, 91.9 cm in maximum width and 30.4 cm in thickness, while the smallest specimen measured 61.8 cm in length, 55.3 cm in width and 17.1 cm in thickness. The analyzed sample, although limited in size, indicate that the cores are relatively small, showing repeated phases (recurrence) of preferential production and rearrangement of the Levallois surface, which is inherent with the re-preparation of both preferential and supplementary platforms.

**Figure 8:**
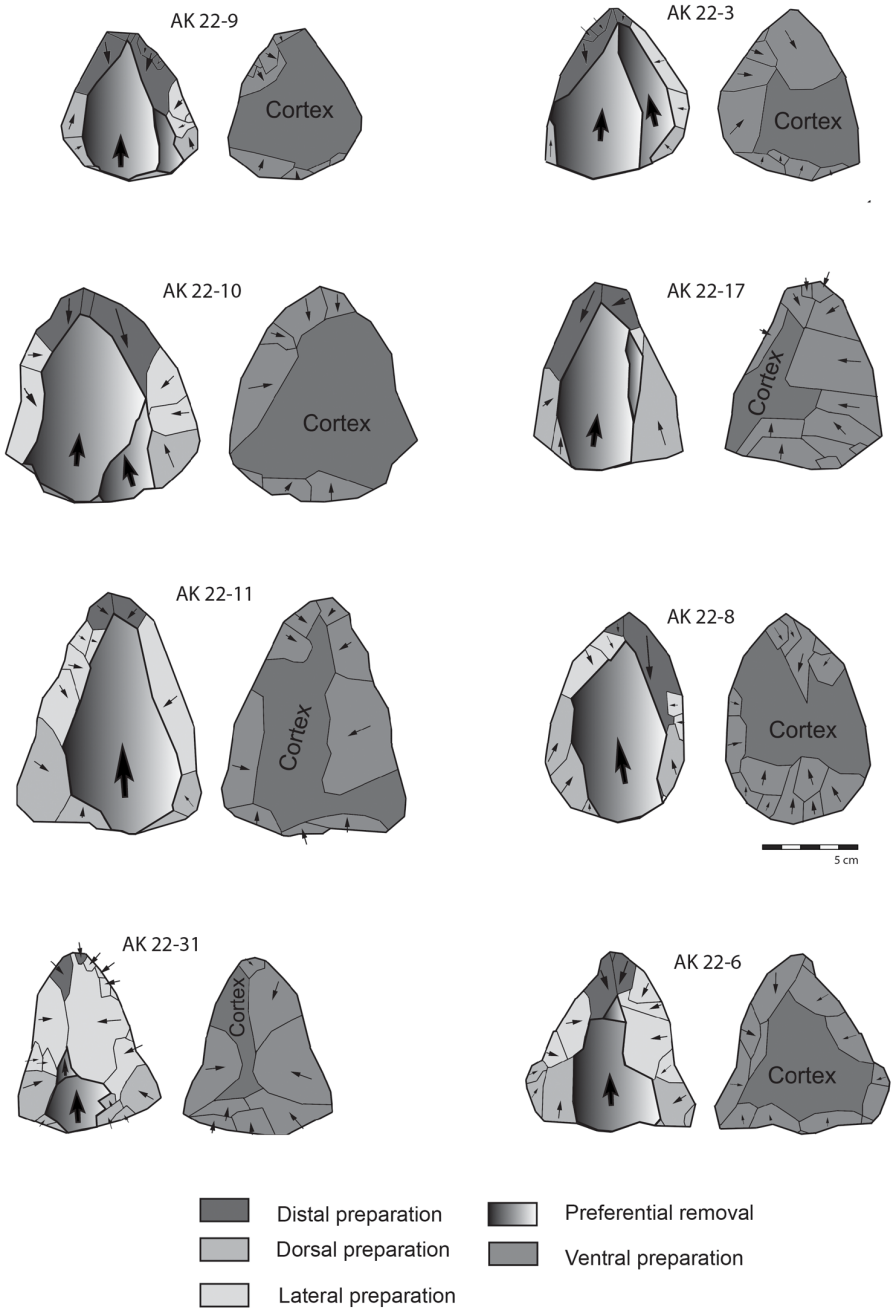
Diacritic schemes showing the directionality of the dorsal and ventral removals on the Nubian core sample from Al-Kharj 22.

**Figure 9:**
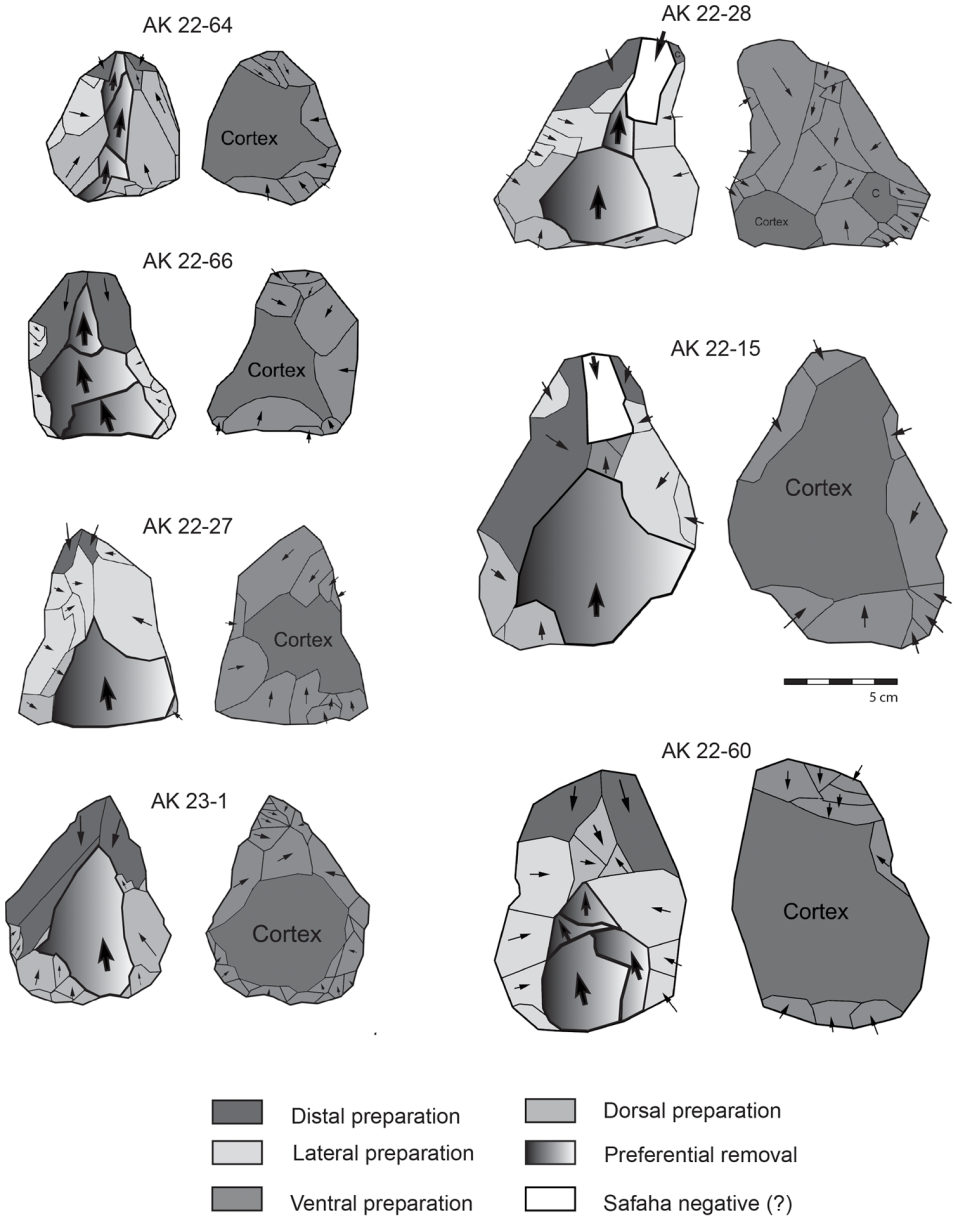
Diacritic schemes showing the directionality of the dorsal and ventral removals on the Nubian core sample from Al-Kharj 22.

Among the analyzed Nubian cores a clear differentiation between ‘preferential’ (primary) proximal platform and the distal (secondary) platform could be made. Thus, platforms placed on the core extremities are managed differently, as the proximal platform is preferential for the extraction of the Levallois triangular flake, while the other serves in the construction of distal ridges and convexities. Lateral preparations on the core's ventral surface were also observed. This feature is found associated with maintenance procedures of the Levallois surface (Figure 8 and Figure 9).

Two of the Nubian cores (Figure 9: cores AK 22–15 and AK 22–28) exhibited elongated scars coming from the distal platform. These removals followed the central axis of the cores hinging at the junction of the distal and medial portion of the Levallois surfaces. These negatives are interpreted as ‘Safaha negatives’ given that the produced blanks would have resembled Safaha blades (*sensu* [70]). These removals exploited the distal to medial guide ridge matching van Peer's description of the Safaha Method. Given the small sample size this discovery remains to be reinforced by further Nubian sites with Safaha occurrences in central Arabia. It is worth noting that the removal did not alter the general triangular shape of the cores, nor did they remove the distal platforms. Given the interpretation by Chiotti et al. [67] for the explanation of this particular technological gesture as a transformation from a Nubian Type 1 core into a centripetal Levallois core, we hereby reject this possibility for the Al-Kharj Nubian sample.

**Figure 10:**
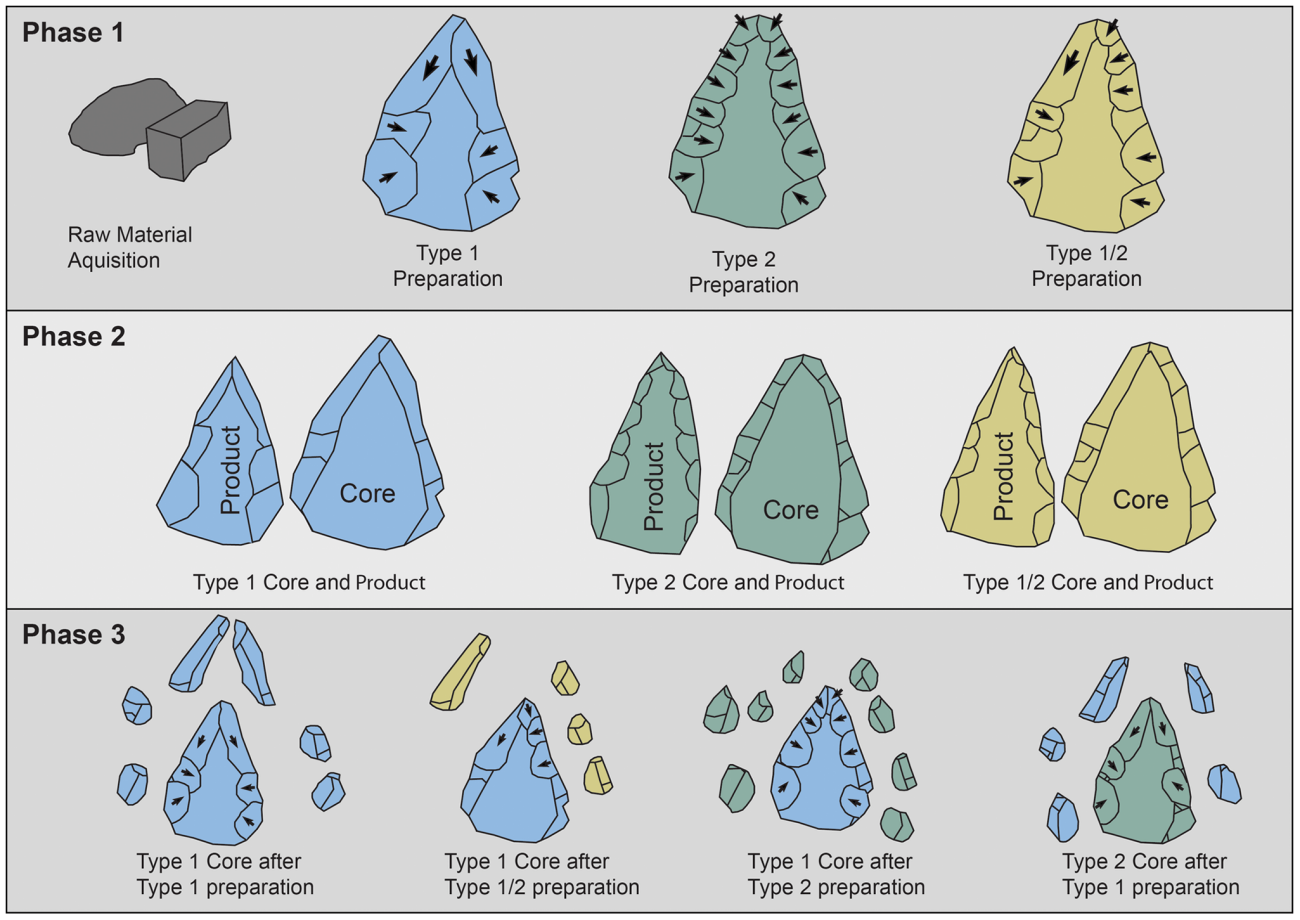
Schematic representation depicting the three main dorsal preparation types, preparation type 1, 2 and 1/2, and the proposed reduction succession discussed in the text. In order to facilitate comprehension cores, end-products and preparation by-products have been color-coded; blue equals type 1 preparation, green type 2 and yellow type 1/2.

Nubian cores may produce more than a single preferential end product when re-preparation takes place. This re-preparation occurred using either a centripetal, distal divergent or a combination of both volume managements strategies. The diacritics coupled with valuable core refittings from Dhofar and North Africa [12], [20], [70], [67] allows for a consolidation of a specific method of core preparation related with the greater Nubian Complex. Therefore, the Nubian reduction strategy (or *chaîne opératoire*/operational sequence, see e.g. [57]) at Al-Kharj 22 is sequenced into the following three main phases (Figure 10).

Phase 1 is marked by the preparation of the platform and Levallois surfaces, this occurs by means of one of the set reduction schema previously identified by various researchers [6], [12], [22], [24], [27], [65], [99]. Either the Levallois preparation takes place through the removal of two distal divergent removals, in which case the preparation of the ventral surface is restricted to the preferential and distal platforms, or it takes place through centripetal removals. In the latter case, the preparation of the ventral surface occurs alternately with the preparation of the Levallois surface. As Guichard and Guichard [65] note, this gives the core the appearance of an asymmetric biface. The parameter for the preference of one preparation method over the other, although yet not fully understood, likely relates to the shape of the raw material. If amorphous nodules prevail within outcrops, the preparation will proceed centripetally. Alternatively, elongated blocks are more likely to be prepared in bidirectional fashion, given that the use of the raw form predetermines the arrangements of the primary and secondary platforms.

In Phase 2, the end product is detached from the prepared Levallois surface. This phase exploits the created convexity altering the Levallois surface in a way that the reduction of a second predetermined blank is impossible without further corrections to the Levallois surface. This does not mean that a complete preparation of the Levallois surface by either modality has to take place.

Phase 3 is marked by either minor modification to the distal convexity by either distal removals or centripetal preparation. The choice of preparation method is interchangeable, in that centripetal, distal and lateral corrections to the Levallois surface are made non-exclusively.

Based on these results and following the suggestion made by Chiotti et al. [67] the following amendments to the definition of the Nubian types are suggested: (a) the Type 1 and Type 2 cores do not represent diverging reduction modalities, rather they are part of the same conceptualization of the Levallois surface; (b) the purely taxonomic classification should be abandoned in favor of a combined techno/typological approach, as has been demonstrated here. This is primarily due to the observation that core reduction is a continuum to which archaeologists only have limited access through a cores typology, given that these only reflect one specific stage within this continuum. This continuum cannot be assessed through metrical variability without a clear understanding of the technological processes involved. Finally, (c) Nubian reduction is not bifurcated into Type 1 or Type 2, but shows adaptation by the flintknapper, driven by negative shapes and obtained convexities acquired after each percussion and is susceptible to change across its continuing reduction. Unlike Chiotti et al. [67], we argue in favor of viewing the Nubian reduction method and the Centripetal Levallois method as separate reductions strategies.

## Discussion and Conclusions

The site of Al-Kharj 22, in the central part of the Arabian Peninsula, provides a new point of reference for regional and interregional comparisons with assemblages from Saudi Arabia, Yemen and Oman. The archaeological investigation of central Saudi Arabia is crucial in terms of establishing comparisons between the Paleolithic records of southern Arabia with its northern provinces. Given the presence of Paleolithic sites in the Al-Kharj region [100], the results presented here greatly expand our knowledge concerning prehistoric occupations within the region and population dispersals across Arabia. Technologically, the Al-Kharj 22 sample exhibits various reduction methods anchored within the recurrent and preferential Levallois methods of which Nubian technology, although not the most numerous, certainly represents a recognizable cultural and technological marker.

The origin of Nubian Levallois technology lies in Africa and is the product of Anatomically Modern Humans [12], [20], [54], [68]. The recognition of two stages within the Nubian Complex at Sai 8-B-11 suggests that the technology emerged in the Nile Valley [68], [99]. Lithic analysis and absolute dating indicates that the Nubian Complex in Africa is partitioned into two phases, an Early and a Late Nubian Complex. The Early Nubian Complex dates approximately to early MIS 5 and is marked by the predominance of Nubian Type 2 cores and lanceolate-shaped bifaces and foliates. The Late Nubian complex, however, dates to the later part of the MIS 5 and lacks the bifacial component presented by the Early Nubian Complex assemblages. Additionally, the Nubian Type 1 core is found more frequently than the Type 2 core [12], [68].

The Arabian Peninsula has, up to this point, presented sites with Nubian technology across the South Arabian Highlands (encompassing the Dhofar Governorate in Oman and the Governorates of Mahra and Hadramawt in Yemen), and the area of Al-Kharj in central Saudi Arabia. Given the absolute dating of Aybut Al Auwal, southern Oman, to a later MIS 5 and the absence of bifacial technology within the Arabian samples (including the South Arabian Highland and the Al-Kharj Area), a general attribution of these assemblages to the Late Nubian Complex from Northeast Africa is possible.

Usik et al. [20] demonstrate that the Afro-Arabian Nubian Complex sites from Dhofar may be partitioned into a Classic Dhofar Nubian industry and a Mudayyan industry. The Al-Kharj 22 assemblage is technologically analogous with the Late Nubian complex in general. The conclusive attribution of the Al-Kharj 22 sample to the Classic Dhofar Nubian industry must remain tentative given the great distance between these two regions and the comparably low number of Nubian cores within the Al-Kharj 22 sample. A possible attribution of the Al-Kharj 22 assemblage to the Mudayyan industry remains inconclusive given the restricted sample size for the Nubian Levallois cores from Al-Kharj 22, but seems unlikely given the presence of large cores at Al-Kharj 22. As such, it is likely that the Al-Kharj 22 Nubian cores belong to a local manifestation of the reduction method developed in North Africa.

A difference between the Nubian assemblages from the South Arabian Highlands and central Saudi Arabia lies in the possible identification of the Safaha Method among the core samples from Al-Kharj 22. This technological gesture remains, up to this point, anomalous among Arabian Nubian Complex assemblages. Unfortunately, the limited sample size for Al-Kharj 22 greatly restricts interpretations and possible chrono/cultural affiliations between this particular aspect of African and Arabian Nubian lithic technology.

The identification of the Nubian reduction methods at Al-Kharj 22 draws general parallels to the South Arabian sites and to the broad and reasonably well-studied regions of the Dhofar and Hadramawt plateaus. The relation between these two regions cannot be explained by technological convergence expressed by two different populations. The presence of Nubian reduction at both regions might relate to population movements between the southern and central portions of the Arabian Peninsula and northeast Africa. Concerning the directionality of the techno-cultural interchanges between Africa and Arabia, it is reasonable to argue in favor of an out-of-Africa movement, given that the Arabian archaeological record lacks a technological predecessor that would have given rise to the Nubian Complex. Geographically, approximately 1000 kilometers stretch between the northernmost occurrence of Nubian technology identified in Dhofar (site TH.38; [12]) and the Al-Kharj 22 site. Population movements across the Arabian Peninsula in a north to south axis are restricted to the following routes: (a) the Red Sea Hills and its coastal environments; (b) across the interior of the Peninsula (e.g. along Jebel Tuwayq); and finally (c) along the ‘Arabo-Persian Gulf oasis’ [101].

Although extensively surveyed [11], [92], [102], [103], southeastern Arabia and the Gulf region failed to produce convincing evidence for Nubian technology [12], thus refuting the use of these regions as a conduit for this particular expansion. Further research will elucidate whether the eastern Nejd Plateau in Dhofar represents the easternmost extent of the Nubian techno-cultural expansion.

The two remaining routes lie in areas that have experienced little to no archaeological investigation, rendering the proposed inferences regarding demographic circulations tentative, at best. The technological similarities observed between the African and the Arabian Nubian Complexes indicate cultural transmission, demographic interaction or diffusion across Arabia and northeast Africa. The palaeoclimatic record for both the Rub' al-Khali desert (the Empty Quarter) and the mountainous and coastal settings of the Red Sea may yet shed some light on the conductivity of these landscapes for Nubian technology bearers. The continually expanding palaeoclimatic record of Arabia, however, suggests that humid episodes across the Peninsula were not spatially uniform; variations in the timing and extent of precipitation incursions from varying sources may mean that favorable environmental conditions could have developed asynchronously between northern, central and southern regions. Indeed, the Red Sea coastal and mountainous environments have been attributed the status of both refugium and corridor [104]–[106]. While the northern part of the Red Sea corridor today receives approximately 180 mm of annual rainfall, the Yemeni western Highlands and the Asir Mountains of Saudi Arabia receive a considerably higher amount of annual rainfall (300–1000 mm). The rain that falls across these regions over the winter months nourishes countless springs and oases along the both westward and eastward facing scarps enabling populations to diffuse from the south to the center of the Arabian Peninsula. Thus, the bearers of the Nubian Complex may have reached Al-Kharj 22 through the riparian systems that connect the Arabian Arch with the Tuwayq Escarpment to the west.

**Figure 11:**
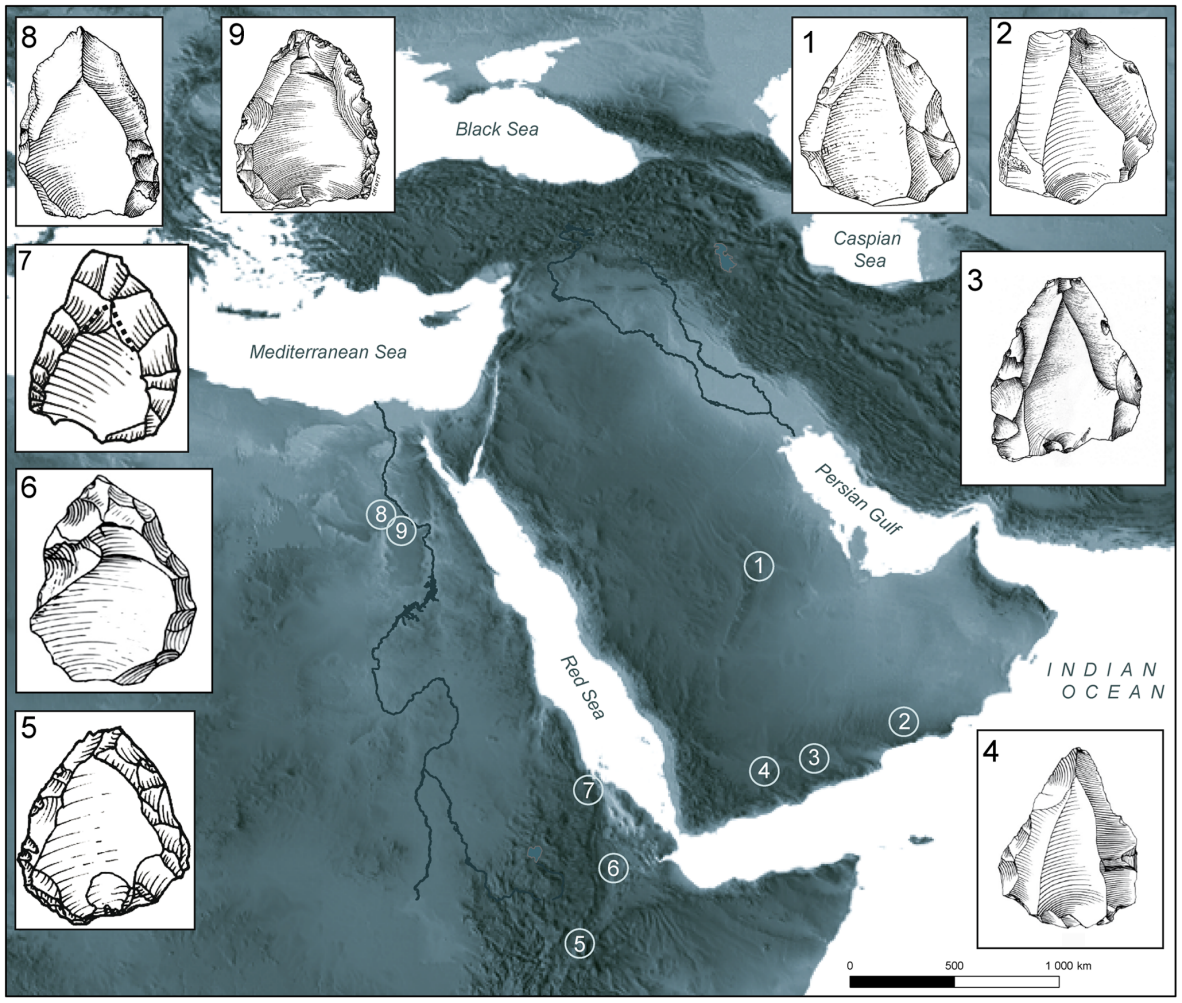
Distribution of main sites with Nubian cores in Eastern Africa and Arabia. Illustrated cores do not represent actual size. 1. Al-Kharj 22 (this study); 2. Aybut Al Auwal [12]; 3. Shabwa [30]; 4. Hadramawt [5], [6], [27]; 5. Aduma [112]; 6. Gademotta [113]; 7. Asfet [114]; 8. Nazlet Khater 1 [115]; 9. Abydos [66].

Alternatively, Nubian technology bearers may have spread across the Rub' al-Khali desert. Relict fluvial and lacustrine records from Saudi Arabia and Oman [107], [108] indicate that large palaeolakes existed during MIS 5 (between ca. 80 and 132 kya), at times coincident with MIS 5e, 5c and 5a. The activation of drainage systems associated with these lakes would have led to the development of a longitudinally-extensive suite of fluvial channels that would have debouched into the Arabian interior. Whilst long-term palaeoenvironmental records from the Rub' al-Khali remain sparse, the presence of large water bodies in areas such as Mundafan [29], [107], support the notion that favorable environmental conditions during MIS 5 would have been conducive to the expansion of human groups into the interior. Unfortunately, little is known about the environmental conditions of the Arabian interior during substages MIS 5d and MIS 5b, however, evidence from speleothem records in Yemen suggest that these periods were typified by increases in aridity [109]. Additionally, a paucity of detailed palaeoenvironmental records between MIS 4 and MIS 2 is indicative of the poor sediment preservation potential of Arabia during phases of increased aridity [85], a shortage of sediment supply due to lower sea levels during mid-high latitude glacial phases, and the stabilization of the Gulf Oasis. Further constraints to dune preservation are re-depositional events, which took place during the Late Glacial Maximum (LGM) [82], [110] and, conversely, erased older environmental records. Therefore, while it remains unclear when the ‘windows of opportunity’ [107], [111] for human expansions across the Rub' al-Khali desert became closed, a growing number of fluvial, lacustrine and speleothem records from Arabia indicate when such favorable climatic windows occurred [74], [75], [107], [108]. The palaeoclimatic record of Arabia indicates that three distinct wet phases occurred during MIS 5 [109]. The first of these wet phases occurred between 130 and 125 kya (MIS 5e) and precedes the presence of Nubian technology in Arabia. The two following wet phases, positioned around 100 kya (MIS 5c) and between 80 to 75 kya (MIS 5a) may be viewed as possible windows for the Nubian expansion into and across Arabia (Figure 11).

The geomorphological observations made during field research indicates that the Al-Kharj area, with its many springs and natural water holes, likely played a role in what may be termed an ‘environmental boon’ during the desiccation following the pluvial phases of MIS 5. This may have allowed the region to become an important refugium, as has been interpreted from other archaeological Upper Pleistocene sites in Arabia [12], [13], [17]. Nubian technology and its associated patterns of core preparation remained part of the North African archaeological record for a considerable timespan. Cores exhibiting Nubian pattern of preparation have been found within Activity phase V and VI at Taramsa 1 [54] alongside variations of volumetric *débitage* and Taramsa blade production systems respectively. Activity Phase V, characterized by a Safahan assemblage has been dated by OSL to between 56.9±6.9 and 39.5±3.8 kya, whilst Activity Phase VI (characterized by an Upper Palaeolithc Late Taramsan type of industry) has yielded ^14^C AMS dates of between 45,348–40,711 (2σ) cal. BP ([54]: table 11.1). Given these circumstances, we remain open to the prospect of Al-Kharj 22 representing a later expansion of Nubian technology into Arabia. Further investigations targeting the discovery of additional Nubian sites in the area will be undertaken in future field campaigns and will hopefully add to our understanding of interregional Nubian technology.

## Materials and Methods

### Ethics

All necessary permits for the Yamama/Al-Kharj fieldwork and analyses were obtained from the Saudi Commission for Tourism and Antiquities, Kingdom of Saudi Arabia.
